# Difference between Primary and Secondary Infertility in Morocco: Frequencies and Associated Factors

**DOI:** 10.22074/ijfs.2018.5188

**Published:** 2018-03-18

**Authors:** Abdelhafid Benksim, Noureddine Elkhoudri, Rachid Ait Addi, Abdellatif Baali, Mohamed Cherkaoui

**Affiliations:** 1 Laboratory of Human Ecology, Department of Biology, School of Sciences Semlalia, Cadi Ayyad University, Marrakech, Morocco; 2 High Institute of Nursing and Technical Health, Marrakech, Morocco; 3 Laboratory of Sciences and Health Technologies, Higher Institute of Health Sciences University Hassan First Settat, Settat, Morocco

**Keywords:** Infertility, Morocco, Women

## Abstract

**Background:**

The main objective of this survey was to determine the difference between primary and secondary infertility in Morocco and the associated factors among women, who are referred to public and private health centers in Morocco.

**Materials and Methods:**

In this cross-sectional study, 619 infertile women referring to public and private health cent- ers in Marrakech-Safi region, were selected by simple random sampling method. This study was conducted between 1 October 2013 and 31 December 2015. Socio-economic status, demographic characteristics, couple’s age, nutritional status and other data related to both male and female reproductive organs were collected by a questionnaire. Logistic regression was used to identify the associated factors to infertility. Statistical significance was set at 0.05.

**Results:**

The rates of primary and secondary infertility were 67.37, and 32.63%, respectively. Multivariate analysis identified a model with three significant predictive factors of secondary infertility: duration of marriage [odds ratio (OR)=12.263: 2.289-65.685], socio-economic status (OR=3.83: 1.011-14.70) and the ages of women (OR=1.268: 1.038-1.549).

**Conclusion:**

The causes of primary and secondary infertility were not always a woman’s problem, but both man and woman contribute to infertility. Multiple regression analysis showed that women’s age, duration of marriage, and socio- economic status are predictive variables that decrease the chance of fertility among women with secondary infertility.

## Introduction

Infertility is a public health problem during the re. productive age, affecting about 10-15% of couplesattempting to achieve pregnancy in worldwide (1). Infertility is defined by the failure to achieve a natu. ral pregnancy after 12 months or more of regular un. protected sexual intercourse (2). For many couples, the inability to bear children is a shocking tragedyleading to serious physical, social, psychological andsexual dysfunction in their lives (3).

According to World Health Organization (WHO), the term primary infertility is used when a womanhas never conceived and secondary infertility is theincapability to conceive in a couple who have had atleast one successful conception in the past (4). Infertility can be attributed to anomalies associated witheither male or female reproductive systems or withboth partners. Several factors can disturb the process of fertility at any step. For example, female infertilitymay be due to one or more reasons such as, polycysticovary syndrome (5), hormonal disorders (6), premature ovarian failure (7), genital infections (8), endo. metriosis (9), fallopian tube obstruction (10), congenital uterine anomalies (11), uterine synechiae (12), or other medical complications (diabetes and thyroiddisorders) (13-14).

Nevertheless, male infertility is due to hormonal imbalances, and sperm abnormalities (3-15). Other main causes of infertility could be age of a couple (16), occupation, and socio-economic status (17). Few studies were dedicated to determine the prevalence and associated risk factors for infertility in Morocco. Therefore, our aim was to determine the difference between primary and secondary infertilities and to better understand the main infertility associated factors in the Moroccan population.

## Materials and Methods

This cross-sectional study was conducted with the approval of the Ethic of the Moroccan health authorities in the region of Marrakech-Safi. This region is located in the middle of Morocco and consists of one state and eight administrative provinces. This study was conducted at different public and private health centers in the region of Marrakech-Safi. A sample of 619 infertile women referring to these health centers was selected by a simple random sampling method, between 1 October 2013 and 31 December 2015. The subjects were chosen without any previous appointments.

The study protocol was explained and the informed consent was obtained from all participants before enrolment. In this study, all data was collected through a questionnaire and the information provided by health booklets for each married woman who had difficulty becoming a mother after at least 12 months of regular unprotected sexual intercourse. The questionnaire contained different elements: socio-economic data, demographic characteristics, age of the couple, and their nutritional status. Also, searching health booklets provided history and clinical information [urogenital infections, medical complications, diabetes, thyroidism, menstrual disorders, polycystic ovary syndrome (PCOS), tubal dysfunction, varicocele and congenital uterine anomalies] and methods of diagnosis, particularly the results of ultrasounds, hysterosalpingographies, hysteroscopies, hormone levels and semen analyses.

Other data associated with male and female reproductive organs were included such as menstruation disorders, uterine malformations, hormonal imbalances, varicocele, the quality of sperms, and other medical complications. 

The subjects who had met our inclusion criteria were married women during their childbearing ages, who had referred to health centers for infertility problems after 12 months of trying for pregnancy. Prior to this research study, the collection tool was tested with a pilot group of women similar to those wishing to participate voluntarily in this survey. All women were entered into the study and their associated data was collected by trained research nursing students.

### Statistical analyses 

A one-sample Kolmogorov-Smirnov test was used to analyze normality for continuous variables. A Chi-square test and Fisher’s exact test were used for categorical variables.

Student’s t test was used to estimate the observed differences between the means. The multivariate data analysis was used to allow for the elimination of the confounding factors and entering the weight of the associated variables with the type of infertility in the bivariate analysis set at 0.2. These variables were used to identify factors that were independently associated with secondary infertility. Associations were measured in odds ratio (OR) with 95% confidence intervals (CI). Data analyses were carried out using SPSS (SPSS Inc. for Windows version 10.0, Chicago). For all analyses the differences were considered significant when P<0.05.

## Results

A total of 619 infertile women were included in this study, 417 (67.37%) with primary infertility and 202 (32.63%) with secondary infertility.

**Table 1:** Comparison of socio-economic and demographic characteristics between primary and secondary infertilities


Variables and modalities		Primary infertility n (%) or Mean ± SD	Secondary infertility n (%) orMean ± SD	P value

Women’s age (Y)		28.7 ± 5.7	31.95 ± 5.6	0.0001
Socioeconomic status				
	Low	28 (6.70)	26 (12.9)	0.003
	Average and high	389 (93.3)	176 (87.1)	
Nutritional status				
	Normal weight	351 (84.2)	172 (85.1)	
	Excess weight	66 (15.80)	30 (14.90)	0.490
Duration of marriage				
	>5 Y	145 (34.8)	146 (72.3)	
	≤5 Y	272 (65.2)	56 (27.7)	0.0001
Period of infertility				
	≤3.8 Y	269 (64.5)	112 (55.4)	
	>3.8 Y	148 (35.5)	90 (44.6)	0.075
Imaging tests (women)				
	Hysterosalpingography (HSG)	163 (39.08)	60 (29,70)	0.023
	Pelvic ultrasonography	394 (94.48)	182 (90,09)	0.033
	Hysteroscopy/ laparoscopy	45 (10.8)	15 (7.4)	0.183
Biological tests (women)				
	Hormonal tests	177 (42.44)	70 (34.65)	0.063
	Post-coital test	14 (3.14)	00 (00)	0.008
Partner age (Y)		35.8 ± 7.7	38.8 ± 6.8	0.001
Partner consultation		239 (57.3)	60 (29.7)	0.001
Semen analysis		237 (56.8)	59 (29.2)	0.001


**Table 2:** Comparison of clinical characteristics between primary and secondary infertilities


Variables and modalities	Primary infertilityn (%)	Secondary infertilityn (%)	P value

Ovulation disorder				
Menstrual disorders	213 (51.1)	92 (45.5)	0.191
Hormone disorder (FSH, LH, AMH)	65 (36.7)	27 (38.6)	0.782
Endocrine diseases (diabetes, thyroid)	22 (5.3)	15 (7.4)	0.290
Polycystic ovary syndrome	107 (27.2)	53 (29.1)	0.620
Tubal factors				
Obstruction and tubal dysfunction	105 (64.40)	41 (68.30)	0.581
Endometriosis/pelvic adhesion				
Endometriosis	14 (8.6)	8 (13.3)	0.290
Uterine synechiae	6 (3.7)	6 (10.0)	0.065
Uterine and cervical factors				
Congenital uterine anomaly	27 (6.9)	15 (8.2)	0.554
Fibroids	25 (6.3)	13 (7.1)	0.721
Polyps	12 (3.0)	4 (2.2)	0.760
Genital infections	65 (15.6)	33 (16.3)	0.810
Male factors				
Varicocele	31 (13.00)	4 (6.7)	0.263
Abnormal sperm	107 (45.1)	12 (20.3)	0.001
Origin of infertility				
Unexplained infertility	20 (8.33)	07 (11.30)	
Male infertility	54 (22.5)	04 (6.50)	0.003
Female infertility	106 (44.20)	41 (66.10)	
Mixed infertility	60 (25)	10 (16.10)	


FSH; Follicle stimulating hormone, LH; Luteinizing hormone, and AMH; Anti-mullerian hormone.

**Table 3:** Variables independently associated with primary (n=202) and secondary (n=417) infertilities, according to the multiple logistic regression model


Variables and modalities	β	χ^2^	P value	OR	95%	CI

Women age	0.237	5.393	0.020	1.268	1.038	1.549
Partner age	-0.129	2.562	0.109	0.879	0.751	1.029
Socio-economic status	1.342	3.848	0.048	3.83	1.011	14.70
Hysterosalpingography	-0.340	0.264	0.608	0.712	0.194	2.606
Post-coital test	-18.499	0.000	0.999	0.000	0.000	-
Partner consultation	-1.817	1.021	0.312	0.162	0.005	5.516
Menstrual disorders	-0.117	0.047	0.829	0.889	0.307	2.574
Uterine synechiae	1.862	2.788	0.095	6.439	0.723	57.314
Abnormal sperm	-0.328	0.321	0.571	0.721	0.232	2.238
Hormone disorder	0.079	0.025	0.875	1.082	0.403	2.904
Duration of marriage: ≥ 5 Y	2.507	8.569	0.003	12.263	2.289	65.685
Period of infertility: ≥3, 8 Y	-1.560	3.628	0.057	0.210	0.042	1.046


OR; Odds ratio and CI; Confidence intervals.

The socio-economic and demographic characteristics between primary and secondary infertilities are presented in Table 1. The average of women’s ages were 28.7 ± 5.7 years and 31.95 ± 5.6 years in primary and secondary infertility, respectively. The average ages of their husbands were 35.8 ± 7.7 years and 38.8 ± 6.8 years in primary and secondary infertility, respectively. The difference between their ages was significant. Also, a good socio-economic situation was reported by primary infertile women (93.3%) compared to those with secondary infertility (P=0.003). After that, the majority of these women reported that their weights were normal and only 15% had excess weight. Furthermore, a longer duration of marriage was reported in secondary infertility compared with primary infertility (P=0.001). 

However, the duration of infertility was not different in the two groups of infertility. In comparison to secondary infertility, primary infertile women showed enthusiasm for medical diagnosis such as hysteronsalpingography (39.08%), and pelvic ultrasound (94.48%). However, in secondary infertility, 70.3% of spouses refused to see a specialist compared with the primary infertility group (P=0.001). The semen analysis was mainly practiced to evaluate primary infertility (56.8%) with a significant difference.

The clinical characteristics between the two groups of infertility were presented in Table 2. With the exception of menstrual disorders, the major causes of primary infertility were entirely due to male reproductive organs, particularly varicocele and abnormalities of semen, with a significant difference. The main causes of secondary infertility were observed mostly among women; such causes include hormonal disturbance, medical complications, polycystic ovary syndrome, tubal dysfunction, genital infections, uterine anomalies, endometriosis and adhesions without significant difference. However, according to multiple logistic regression models, variables independently associated with primary and secondary infertility, are presented in Table 3. In this model, the duration of marriage (OR= 12.263: 2.289-65.685), the age of the woman (OR=1.268: 1.038-1.549), and the socio-economic status (OR=3.83: 1.011-14.70) were relatively independent predictive variables associated to secondary infertility. 

## Discussion

To our knowledge, this is the first study able to determine the difference between primary and secondary infertility in Morocco and to distinguish among the associated factors. The overall rates of primary and secondary infertility were 67.37 and 32.63%, respectively. This result is similar to those published in other areas of Africa (18). In secondary infertility, the couple’s average age was higher when compared to those who had primary infertility. Previously, this age difference has been highlighted by other researchers to some degree (17).

In this study, this result can be explained partly by a major change in the age for marriage in Morocco (from 17.3 years old in 1960 to 26.6 years old in 2010) (19). The age of couple is clearly an important factor in reproduction, as a woman’s fertility is strictly dependent on age. The peak of ability to reproduce usually around the age of 20 years for women. Indeed, it starts to decline from the age of 30, and reduce severely from the age of 40 (16).

In secondary infertility, an overly long duration of marriage and an advanced age of the couple could decrease their chances of having a new child. This finding was close to the one recorded by Keskin et al. (17). Also, the socio-economic level of a couple can influence the type of their infertility.

In primary infertility, the majority of women with relatively high-to-moderate socio-economic status are able to resolve their infertility problems. This status can provide fast and easy access to several diagnostic methods and infertility treatments (21). Moreover, excess body weight of women was a powerful determinant of infertility risk by ovulation disorders (22). 

According to the perception of the participants, however, their body weights were normal in both primary and secondary infertility groups. This perception is not compatible with that observed among Moroccan population (23). Also, the apparent weight does not always reflect actual weight status based on the body mass index (24). In fact, the diagnosis of male infertility is an essential step for a quick and effective treatment. However, the sperm abnormality is the major cause for infertility (45.1%) among primary infertile men compared to secondary infertility (20.3%).

This result can be explained by the advanced age and also exposure to urogenital infections affecting the quantity and the quality of sperms. In Africa, the sperm abnormalities were estimated at 68% for the age 31-40 years (15). In this investigation, the rate of female infertility was significantly higher in secondary infertility than primary (66.10 vs. 44.20%). Also, the most common causes of female infertility were ovulation disorders, which manifest themselves by sparse or absent menstrual periods (22). Furthermore, certain studies have demonstrated that 40 to 50% of infertilities were due to female reproductive organs (21).

Finally, the difference between primary and secondary infertilities in Morocco was relatively associated with three independent variables, particularly the duration of marriage (OR=12.263: 2.289-65.685, the woman’s age (OR=1.268) and socio-economic position (OR 3.83). Furthermore, the bounds of this interval were very far from the value 1, which means that the result was positive and therefore the duration of infertility was strongly related to secondary infertility. 

To reduce the limitations in our study, further investigations should be undertaken with control groups of fertile women to provide additional information on risk factors for male and female infertility. Furthermore, a high-quality dialogue between all participants will be recommended for better management of infertility.

## Conclusion

In this study, primary and secondary infertilities were due to the intersection of several demographic characteristics and medical factors. However, woman’s age, duration of marriage and socio-economic status had a significant impact to accentuate the severity of secondary infertility.
